# Spatial–temporal distribution and key factors of urban land use ecological efficiency in the Loess Plateau of China

**DOI:** 10.1038/s41598-023-49807-6

**Published:** 2023-12-15

**Authors:** Lanyue Zhang, Yi Xiao, Yimeng Guo, Xinmeng Qian

**Affiliations:** 1 School of Digital Economics, Sichuan University Jinjiang College, Meishan, 620860 Sichuan China; 2 Business School, Chengdu University of Technology, Chengdu, 610059 Sichuan China; 3 Department of Economics, Sichuan Institute of Administration, Chengdu, 610071 Sichuan China; 4 College of Management Science, Chengdu University of Technology, Chengdu, 610059 Sichuan China

**Keywords:** Environmental sciences, Environmental social sciences

## Abstract

Urban land use ecological efficiency is crucial to the urbanization process and urban ecosystem sustainability. Cities in ecologically sensitive zones with frequent natural disasters need more complex land use patterns and plans. Achieving the goal of harmonizing economy and ecosystem is key for sustainable development policy makers in these cities. Aiming to explore the urban land use ecological efficiency (LUEE) of ecologically sensitive areas, urban land use ecological efficiency index system of the Loess Plateau was constructed, the SBM-Tobit model was adopted to measure the LUEE and influencing factors from 2009 to 2018, and the characteristics of spatial–temporal evolution was discussed. The results indicated that there were significant spatial differences of LUEE in ecologically sensitive zone. The high-level cities of LUEE were located in the southwest areas, while low-level cities of LUEE were mostly situated in the northeast zones, and the temporal variation trend showed the characteristic of “W” curve. Additionally, the results of key factors identification demonstrated that science and technology expenditure and public transport development had positive effects on urban LUEE, while the land expansion, GDP growth, the second industry and real estate development will limit the improvement of urban LUEE. This study used the scientific evaluation index system and key factors identification method to explore the land use ecological efficiency in ecologically sensitive zones, aiming to provide a case study reference for urban land management and optimization in ecologically fragile areas.

## Introduction

With the development of social productivity, the global urbanization process has accelerated since the industrial revolution^1,2^. The speed of land expansion is faster than population urbanization, which aggravates the contradiction between land system and ecosystem, and seriously hinders the harmonious relationship between human and nature^3,4^. The increase of population base and expansion of the scope of human production and living have led to a sharp rise in the social demand for land resources in urban development. The land is historical product of natural processes, the carrier for human survival and development, and indispensable resources for economic development as well as progress of human society. However, the land has gradually become a scarce resource with the deepening of urbanization process. As a complex system involved in the process of production and living, the current situation of urban LUEE reflects the differences of the basic form between layout planning and functional areas, and directly affects the urban socioeconomic system and ecological system^5^. Therefore, how to protect and use land resources sustainably in the process of production and living is the key issue, which is essential to the urban planning and land protection.

Under the new development pattern of China, it is particularly important to coordinate the shortage of resource elements and allocate resource elements rationally. Soil erosion, landslide disaster, barren land and drought directly lead to the limitation of land resources for sustainable use on the Loess Plateau. Rapid urbanization and industrialization will also occupy ecological protection land and agricultural land. These problems restrict urban green space, sustainable agriculture development and livable city construction. The urban land use ecological efficiency refers to the coordinated improvement of economic, social and environmental benefits in the process of urban land use, taking ecological protection as the premise and efficient use of land resources as the goal. It embodies the harmonious symbiosis between human activities and the natural environment, and is an important indicator to measure the level of sustainable development of cities. The urban land use ecological efficiency emphasizes the balance and coordination in urban planning, land resource allocation, ecological protection and environmental quality, aiming at the maximization of land resource utilization, the minimization of environmental pollution, and the sustainable development of social economy^6,7^. Exploring urban LUEE in the study area is conducive to urban planning and ecological protection in ecologically fragile areas. How to evaluate urban land use ecological efficiency scientifically and reveal the key factors is crucial to city sustainability and environmental protection, especially in underdeveloped areas with scarce land resources and frequent natural disasters. The differences between the geographical location, ecological environment and industrial structure of the cities show spatial non-stationarity over time, thus leading to spatial differences in LUEE at various stages of urban development.

The existing researches have studied land use in different regions and industries^8,9^. In terms of land-use types, urban LUEE is related to the development degree of economic zones, including the development time and scale^10^. Urban land-use types are more complex than those in rural areas, urban land use is characterized by diversification, while rural land use is with fragmentation. In terms of economically developed areas, some studies have suggested that fragmentation and diversity of land use are the key factors that distinguish urban and rural areas. Generally, the land use of urban areas is the most diversified, while those in rural areas are the least diversified^11^. Rural LUEE and agricultural scale complement each other, the improvement of LUEE is conducive to the enhancement of agricultural planting scale and agricultural economic benefits^12^. The optimization level of industrial land is relatively high, showing obvious regional and industrial differences. Therefore, appropriate industrial transfer is beneficial for improving urban LUEE^13^. Existing studies have found that there is a close relationship between urban socioeconomic development and urban LUEE. The acceleration of urbanization will put pressure on the urban LUEE, balancing the relationship between urbanization and land use is beneficial for promoting efficient urban development^14^. The new development pattern in the new era prompts scholars to explore the harmonious relationship between land use and ecological environment protection, and the researches on the efficiency of green space utilization is gradually taken seriously^15,16^.

Presently, the researches on urban LUEE focus on the differences between economic development and economic structure, it could be found that cities with high LUEE are generally located in regional economic growth center^17^. Transportation infrastructure construction is also an important factor affecting urban LUEE. Analyzing the relationship between traffic convenience and LUEE can optimize the urban traffic network, which is conducive to the circulation of resource elements^18^. Government regulatory measures directly affect the use and development of land resources, and the local government's implementation of relevant land use policies will also lead to changes in LUEE, which will be affected by land tax policies and prices^19^. Additionally, heterogeneous urban form and layout will also have an impact on urban LUEE, patch size and edge density have different effects on LUEE of large, medium, and small cities^20^.

In the existing studies, the methods of evaluating urban LUEE focus on DEA^21,22^, the semi-parametric estimation method^23^, SBM model^24,25^, and SFP^26^. Existing studies have used these methods to construct the evaluation indexes of urban LUEE. The existing researches widely adopt the multi-index evaluation method to analyze the comprehensive level of urban LUEE from the aspects of ecology, industry, government and economy, and these factors are mainly divided into output factors and input factors^27,28^. From what has been discussed above, it could be concluded that existing researches mainly adopt these methods to measure urban LUEE and construct the evaluation index system. Presently, most of the researches are aimed at urban agglomerations in various countries and representative provinces with high economic development level, lacking investigations on urban LUEE and its key factors in ecologically sensitive areas. The DEA model and SBM model are used in most investigations.

On this basis, SBM and Tobit models are used in this study, which make up for the shortcomings of traditional DEA models in dealing with relaxation variables, and can effectively evaluate the efficiency of unexpected output. By building an indicator system for evaluating the urban LUEE in the study area, the thirty-nine cities are selected for investigation from 2009 to 2018, and the corresponding policy suggestions to improve the LUEE of 39 cities are put forward based on the analysis of key factors.

## Study area and methodology

### Study area

**Figure 1:**
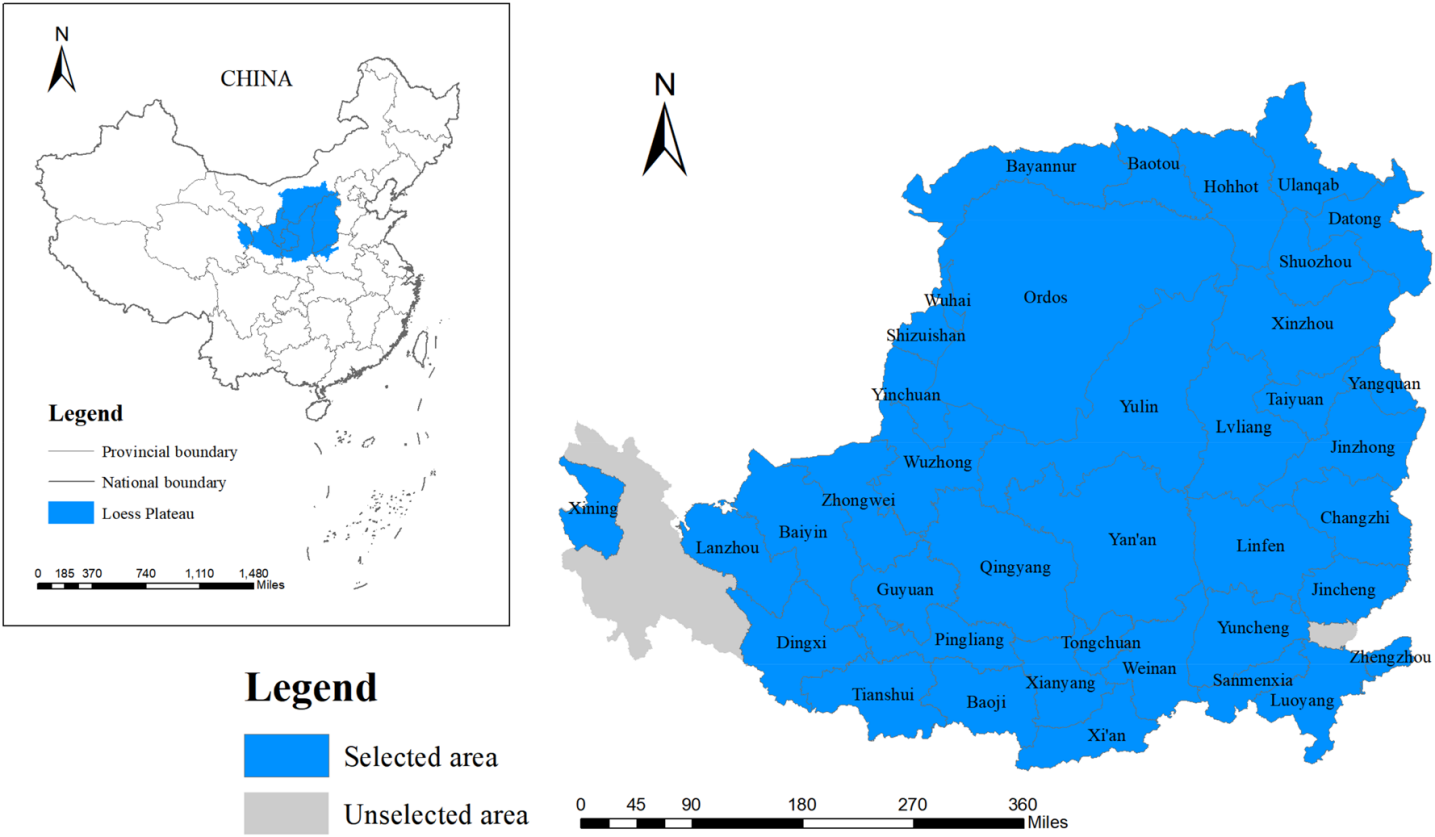
Geographical location of the Loess Plateau, China (Mapping based on the ArcGIS10.8 software can be obtained from the following link, https://desktop.arcgis.com).

The Loess Plateau belongs to the arid continental monsoon climate region. The precipitation is relatively small and the temporal and spatial distribution is uneven. In addition, the loess is of strong water sensitivity due to its large pores and rich carbonate content. Under the influence and threat of extreme weather, rainstorms occur frequently in the Loess Plateau, causing a variety of natural disasters and hindering the sustainable development of areas. With the gathering of urban residents, the continuous reclamation of wasteland by residents to meet the social development demand has led to the destruction of the original vegetation and intensified the soil erosion in this area. The continuous expansion of urban functional areas has significantly increased regional ecological risks, leading to the shortage of land resources and prominent ecological degradation. This paper excludes cities where data cannot be obtained, and considers the integrity of administrative divisions, selecting 39 cities to explore land use ecological efficiency and its key factors (Fig. 1).

### SBM model

Compared with the stochastic frontier analysis method, which needs to set the production function as the premise, the DEA method is more convenient. However, the traditional data envelopment analysis method cannot effectively solve the problem of input–output relaxation. In the actual production and urban construction, output factors include expected factors and non-expected factors. Therefore, the improved SBM model containing undesired outputs is proposed. The specific formula is as follows.

1 $$ \rho^{ * } = \min \frac{{\frac{1}{m}\sum\limits_{i = 1}^{m} {\frac{{x_{i}^{ - } }}{{x_{io} }}} }}{{\frac{1}{{s_{1} + s_{2} }}\left( {\sum\limits_{\gamma = 1}^{{s_{1} }} {\frac{{\overline{{y_{\gamma }^{g} }} }}{{y_{\gamma o}^{g} }}} + \sum\limits_{l = 1}^{{s_{2} }} {\frac{{\overline{{y_{l}^{b} }} }}{{y_{lo}^{b} }}} } \right)}} $$

2 $$ s.t.\left\{ \begin{gathered} \overline{x} \ge \sum\limits_{j = 1, \ne 0}^{n} {\lambda_{j} x_{j} ,\quad j = 1, \ldots ,m} \hfill \\ \overline{{y^{g} }} \le \sum\limits_{j = 1, \ne 0}^{n} {\lambda_{j} y_{j}^{g} ,\quad r = 1, \ldots ,s_{1} } \hfill \\ \overline{{y^{b} }} \ge \sum\limits_{j = 1, \ne 0}^{n} {\lambda_{j} y_{j}^{b} ,\quad l = 1, \ldots ,s_{2} } \hfill \\ \overline{x} \ge x_{o} ,\;\overline{{y^{g} }} \ge y_{o}^{g} ,\;\overline{{y^{b} }} \ge y_{o}^{b} ,\;\lambda \ge 0,\;\sum\limits_{i = 1, \ne 0}^{n} {\lambda_{j} = 1} \hfill \\ \end{gathered} \right. $$

where $x$, $y^{b}$ and $y^{g}$ represent input, expected output, and undesired output respectively, $s^{ - }$, $s^{b}$, and $s^{g}$ represent relaxation variables of input, expected output, and undesired output respectively, $\lambda$ is the weight vector and $\lambda \ge 0$. $X$, $Y^{g}$ and $Y^{b}$ are matrices, $X = \left[ {x_{1} , \ldots ,x_{n} } \right] \in R_{m \times n}$, $Y^{g} = \left[ {y_{1}^{g} , \ldots ,y_{n}^{g} } \right] \in R_{{s_{1} \times n}}$, $Y^{b} = \left[ {y_{1}^{b} , \ldots ,y_{n}^{b} } \right] \in R_{{s_{2} \times n}}$. $m$, $s_{1}$, and $s_{2}$ represent the number of input factors, expected output and undesired output indicators respectively. The subscript “$o$” in the lower right corner of the variable indicates the evaluated decision-making unit. The attribute and interval of $\rho^{ * }$ are the same as formula (1), but when the efficiency value of the model $\rho^{ * } = 1$, which cannot distinguish the effective DMUs. The $\rho^{ * }$, $x$, $y^{b}$, $y^{g}$, and $\lambda$ are the same as those in formula (1) and formula (3), the “” above the variable represents the projection value.

### Tobit model

Due to the SBM model cannot research the influencing factors of urban LUEE in the Loess Plateau, Tobit model was adopted for discussion. The basic model is as follows:

3 $$ Y_{i} = \alpha_{i} + \sum\limits_{i = 1}^{n} {\beta_{i} X_{it} + \varepsilon_{it} } $$

where $Y_{i}$ refers to the explained variable, and the evaluation value of urban LUEE is the explained variable of this research. $\alpha_{i}$ represents the intercepted item, $\beta_{i}$ is the parameters of the item, $X_{it}$ represents the explanatory variable. $\varepsilon_{it}$ represents the random perturbation term.

Based on the discussion of the influential factors of urban LUEE in previous studies, it could be found that land use mode, population and economic growth, infrastructure construction, environmental protection and fixed asset investment are the key factors affecting land use ecological efficiency^29–31^. This study used the Pearson correlation coefficient to measure the linear correlation between influencing factors, and the value range of Pearson correlation coefficient is [− 1, 1]. The greater the absolute value of Pearson correlation coefficient, the higher the linear correlation between the variables, and the coefficient value of 0 means that there is no linear relationship between variables. The variables tested by Pearson correlation coefficient are shown: natural population growth rate (*PG*), proportion of urban construction land in urban area (*PCL*), regional GDP growth rate (*GG*), proportion of secondary industry (*PS*), the number of buses per 10,000 people (*NBP*), centralized treatment rate of sewage treatment plant (*CTR*), urban real estate investment (*REI*), the proportion of R&D expenditure in government (*PRE*). The specific calculation formula is as follows:

4 $$ \begin{aligned} Y_{it} & = \alpha_{i} + \beta_{1} \ln \left( {PG_{it} } \right) + \beta_{2} \ln \left( {PCL_{it} } \right) + \beta_{3} \ln \left( {GG_{it} } \right) + \beta_{4} \ln \left( {PS_{it} } \right) \\ & \quad + \beta_{5} \ln \left( {NBP_{it} } \right) + \beta_{6} \ln \left( {CTR_{it} } \right) + \beta_{7} \ln \left( {REI_{it} } \right) + \beta_{8} \ln \left( {PRE_{it} } \right) + \varepsilon_{it} \\ \end{aligned} $$

**Figure 2:**
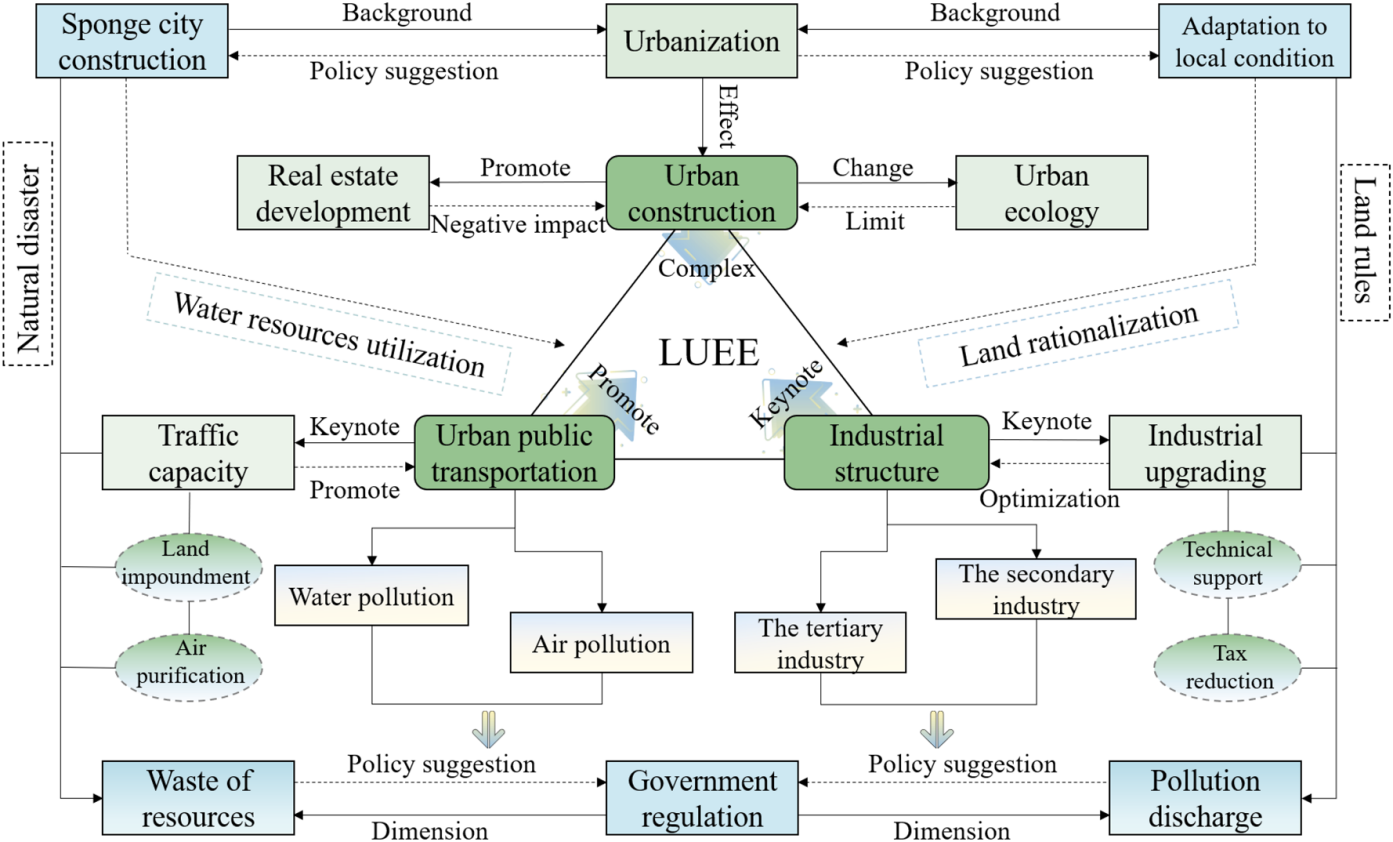
The analysis framework of this study.

From what has been discussed above, the LUEE of 39 cities in the study area was empirically analyzed and relevant policy recommendations were proposed. The specific analysis path is shown in Fig. 2.

### Evaluation index system

By summarizing and refining the existing researches, it could be concluded that the index system is divided into two aspects: expected output elements, and undesired output elements^32–36^. The indicator “Urban construction land area” was selected to measure the input condition of land resources in urban construction. “Total fixed asset investment” was adopted to represent the urban total capital factor input, the input capital indicator was calculated using the perpetual inventory method to get the urban capital stock based on 2009, and the depreciation rate is calculated at 9.6%. The indicator “Urban employed population” was chosen to evaluate the number of urban labor force. The expected outputs of urban construction include economic growth, social progress and welfare growth, and urban environment improvement^37,38^. The "Per capita GDP" indicator evaluates the current situation of urban development from the economic perspective, selecting “Afforestation coverage rate of built-up area” to measure the urban environmental governance capacity. Undesirable output refers to the adverse impact on the ecological environment and social progress caused by land use, including air pollution, soil pollution, water pollution, and other obstacles hindering the high-quality development of cities^39,40^. This paper selects some ecological environment pollution indicators caused in the process of land use for research. The evaluation index system of urban LUEE was shown in Table 1.

**Table 1:** The evaluation index system of urban LUEE.

Elements	Primary indicator	Indicators	Attribute
Input indicators	Land	Urban construction land area	+
	Capital	Total fixed asset investment	+
	Labor	Urban employed population	+
Expected output	Economy	Per capita GDP	+
	Society	Average wage of employees	+
	Ecological	Afforestation coverage rate of built-up area	+
Undesired output	Environment pollution	Emissions of industrial wastewater	−
		Emissions of industrial SO_2_	−
		Emissions of industrial smoke (dust)	−

### Data sources

The data of this study area includes land use, ecological environment, and socioeconomic development. Urban land use ecological efficiency data refers to the variables related to urban construction and planning. The data sources include *China Urban Statistical Yearbook (2010–2019), Provincial and Municipal Statistical Yearbooks (2010–2019), Provincial and Municipal Ecological Environment Bulletin (2010–2019)*, and some unavailable data were supplemented by interpolation*.*

The existing studies on urban land use ecological efficiency and urban development efficiency have concluded that population factors, economic structure, urban construction and government decisions are closely related to urban LUEE^17,41^. Based on correlation tests and data availability of variables, eight variables were selected in this study, which is shown in Table 2.

**Table 2:** Variable descriptive statistical results.

Variable	Unit	Observations	Maximum value	Minimum value	Range	Standard deviation
Natural population growth rate (*PG*)	%	390	22.81	− 15.90	38.71	4.32
Proportion of urban construction land in urban area (*PCL*)	%	390	52.38	0.36	52.02	10.11
Regional GDP growth rate (*GG*)	%	390	38.90	− 5.56	44.46	4.80
Proportion of secondary industry (*PS*)	%	390	401.12	34.98	366.15	69.65
The number of buses per 10,000 people (*NBP*)	Car	390	29.25	1.13	28.12	5.02
Centralized treatment rate of sewage treatment plant (*CTR*)	%	390	100.00	18.30	81.70	16.51
Urban real estate investment (*REI*)	Yuan	390	2877.15	1.73	2875.42	370.38
The proportion of R&D expenditure (*PRE*)	%	390	0.05	0.00	0.05	0.01

## Results

### Measurement results of urban LUEE

**Figure 3:**
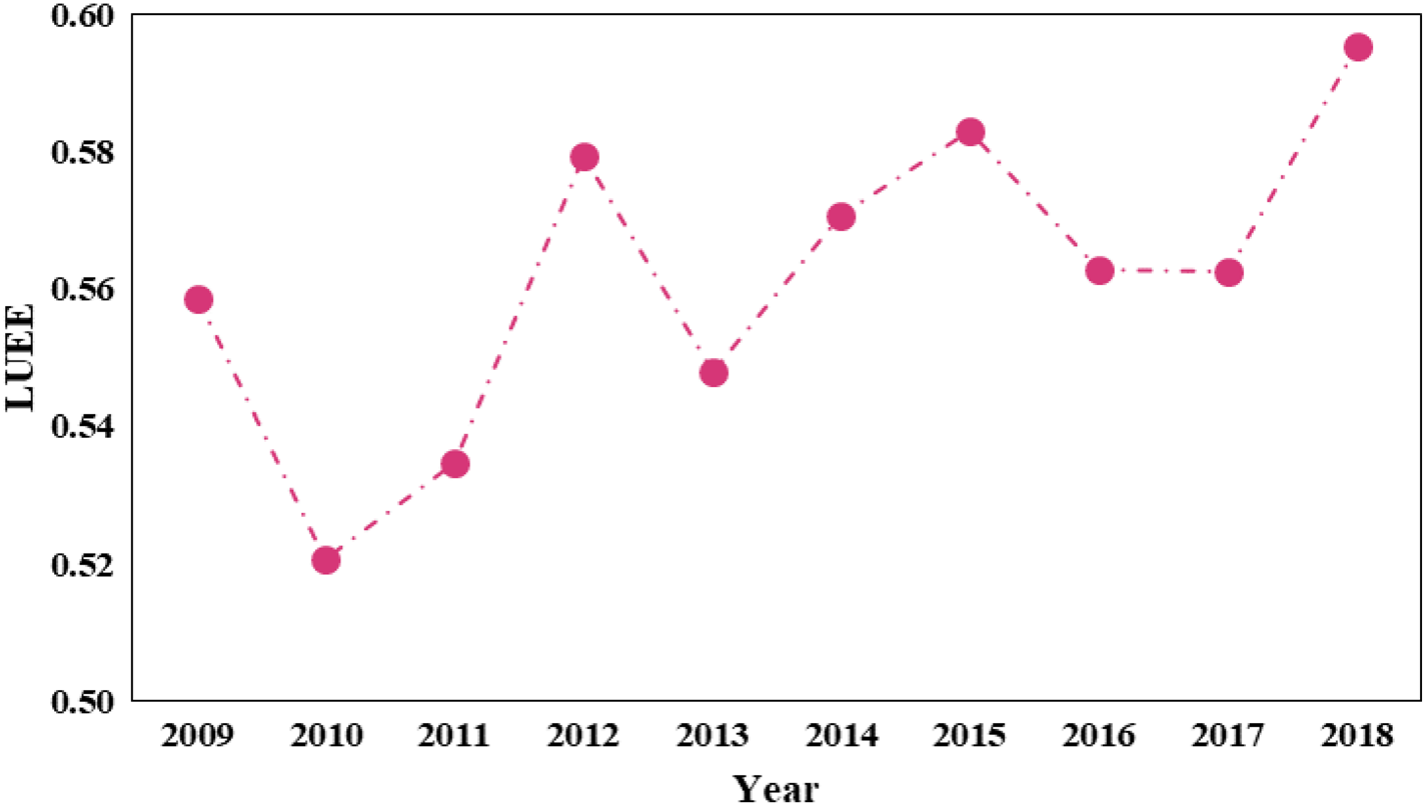
Temporal evolution of urban LUEE in the study area from 2009 to 2018.

Based on the MaxDEA8.0 software and SBM model, the comprehensive levels of urban LUEE of this area from 2009 to 2018 were calculated (Table 3). The results showed that from 2009 to 2018, the temporal evolution of urban LUEE on the study area presented a "W" upward trend curve (Fig. 3). The study found that the urban LUEE of the cities in this region was obviously different, Luoyang had the lowest average level of urban LUEE Guyuan had the highest average value of urban LUEE. Although the comprehensive level of urban LUEE in the study area showed a dynamic growth trend, the growth rate was not ideal. There was a downward trend from 2009 to 2010, 2012 to 2013, and 2015 to 2016, indicating that the urban and use ecological efficiency fluctuated obviously in the Loess Plateau, which was mainly influenced by socioeconomic activities and natural environment.

**Table 3:** Measurement results of urban LUEE in the Loess Plateau.

City	2009	2010	2011	2012	2013	2014	2015	2016	2017	2018	AVG
Taiyuan	0.120	0.122	0.114	0.113	0.141	0.133	0.138	0.145	0.180	0.265	0.150
Datong	0.216	0.210	0.219	0.237	0.253	0.262	0.199	0.208	0.211	0.190	0.221
Yangquan	0.600	0.545	0.571	1.028	0.389	1.016	1.021	0.492	1.007	1.004	0.786
Changzhi	0.344	0.315	0.282	0.304	0.325	0.297	0.282	0.281	0.192	0.194	0.274
Jincheng	0.454	0.468	0.478	0.481	0.577	0.473	0.473	0.420	0.330	0.441	0.460
Shuozhou	1.168	1.174	1.157	1.147	1.060	1.160	0.724	1.092	1.122	1.275	1.101
Jinzhong	0.301	0.299	0.310	0.283	0.301	0.302	0.271	0.223	0.228	0.299	0.279
Yuncheng	0.203	0.211	0.208	0.244	0.325	0.272	0.307	0.282	0.289	0.308	0.272
Xinzhou	0.323	0.263	0.271	0.288	0.389	0.313	0.313	0.298	0.351	0.420	0.323
Linfen	0.282	0.369	0.300	0.249	0.314	0.261	0.267	0.245	0.237	0.219	0.273
Lvliang	1.072	1.047	1.048	1.045	1.013	1.039	1.016	1.021	1.038	1.044	1.034
Hohhot	0.300	0.234	0.279	0.316	0.223	0.191	0.298	0.179	0.206	0.188	0.235
Baotou	0.245	0.208	0.180	0.181	0.336	0.207	0.217	0.273	0.275	0.173	0.228
Wuhai	1.068	1.053	1.038	1.052	1.117	1.124	1.132	1.104	1.092	1.108	1.091
Ordos	1.032	1.067	0.491	0.421	0.334	0.348	1.008	0.365	0.281	0.377	0.521
Bayannur	0.373	0.377	0.455	0.537	0.640	0.468	0.521	0.461	0.412	0.411	0.476
Ulanqab	0.396	0.389	0.369	0.563	0.400	1.065	0.463	0.460	0.400	0.425	0.504
Zhengzhou	0.066	0.060	0.055	0.059	0.078	0.159	0.055	0.063	0.061	0.084	0.075
Luoyang	0.115	0.100	0.095	0.113	0.118	0.206	0.122	0.121	0.127	0.166	0.130
Sanmenxia	0.607	0.532	0.432	0.605	0.525	0.302	1.010	0.434	0.469	0.521	0.537
Xi'an	0.062	0.068	0.063	0.073	0.109	0.069	0.099	1.099	1.030	1.026	0.404
Tongchuan	1.160	1.145	1.248	1.232	1.181	1.166	1.202	1.104	1.016	1.005	1.144
Baoji	0.197	0.224	0.412	0.520	0.421	0.350	0.350	0.264	0.341	0.478	0.373
Xianyang	0.177	0.185	0.172	0.233	0.280	0.253	0.286	0.297	1.140	1.197	0.449
Weinan	0.221	0.257	0.234	0.253	0.260	0.273	0.233	0.182	0.196	0.208	0.233
Yan'an	1.091	1.049	1.008	1.057	1.091	0.582	1.041	1.007	0.422	0.569	0.870
Yulin	0.197	0.226	0.201	0.236	0.255	0.235	0.300	0.274	0.333	0.426	0.276
Lanzhou	0.132	0.140	0.116	0.127	0.162	0.168	0.157	0.171	0.162	0.169	0.152
Baiyin	0.491	0.492	0.507	1.021	1.160	1.009	1.072	1.036	1.532	1.021	0.983
Tianshui	1.001	0.503	1.053	1.057	1.107	1.075	1.096	1.040	0.477	1.114	0.947
Pingliang	0.374	0.092	0.074	0.099	0.098	0.554	0.352	1.006	0.454	0.485	0.357
Qingyang	1.125	1.106	1.294	1.244	1.139	1.334	1.336	1.367	1.149	1.229	1.244
Dingxi	1.104	1.122	1.155	1.218	1.237	1.004	1.062	0.677	1.013	1.008	1.055
Yinchuan	0.406	0.401	0.245	0.335	0.334	0.239	0.272	0.297	0.241	0.356	0.302
Shizuishan	1.047	0.590	1.018	1.007	0.049	0.550	0.693	0.627	0.679	0.556	0.641
Wuzhong	1.033	1.055	1.076	1.025	1.003	1.027	0.596	0.578	0.534	0.558	0.828
Guyuan	1.199	1.228	1.270	1.150	1.211	1.258	1.316	1.397	1.347	1.356	1.281
Zhongwei	1.107	1.055	1.062	1.132	1.088	1.170	1.125	1.022	1.077	1.101	1.092
Xining	0.379	0.332	0.296	0.311	0.326	0.345	0.318	0.342	0.285	0.249	0.311

In 2009, Guyuan had the highest evaluation value of urban LUEE (1.199), Xi'an had the lowest evaluation value of urban LUEE (0.062), the difference in LUEE value between these two cities was 1.137. In terms of 2018, the difference between Guyuan (1.356) with the highest LUEE evaluation score and Zhengzhou (0.084) with the lowest LUEE evaluation score was 0.084, which was higher than that in 2009. It could be found from the average results that thirty-one cities on the Loess Plateau have not achieved effective allocation of urban land use ecological efficiency, accounting for 79.49%. The possible reason is that the current ecological environment protection and ecological restoration strategies still need to be further strengthened, and the local government should implement more efficient land governance and management planning strategies.

From the change trend of a single city, the LUEE of 19 cities were declining. Indicating that nearly half of cities in the study area were not properly allocated in land use, which resulted in insufficient utilization of land resources in urban planning and construction. There are four cities with significantly decreased trends in LUEE, including Ordos, Yan'an, Shizuishan, and Wuzhong. The comprehensive level of LUEE in Ordos decreased from 1.032 in 2009 to 0.377 in 2018, Yan'an decreased from 1.091 in 2009 to 0.569 in 2018, Shizuishan decreased from 1.047 in 2009 to 0.556 in 2018, Wuzhong decreased from 1.033 in 2009 to 0.558 in 2018. Besides, there were three cities with high evaluation levels of LUEE from 2009 to 2018, including Zhongwei, Guyuan, and Qingyang, which shows that the LUEE of these cities were at a high level.

**Figure 4:**
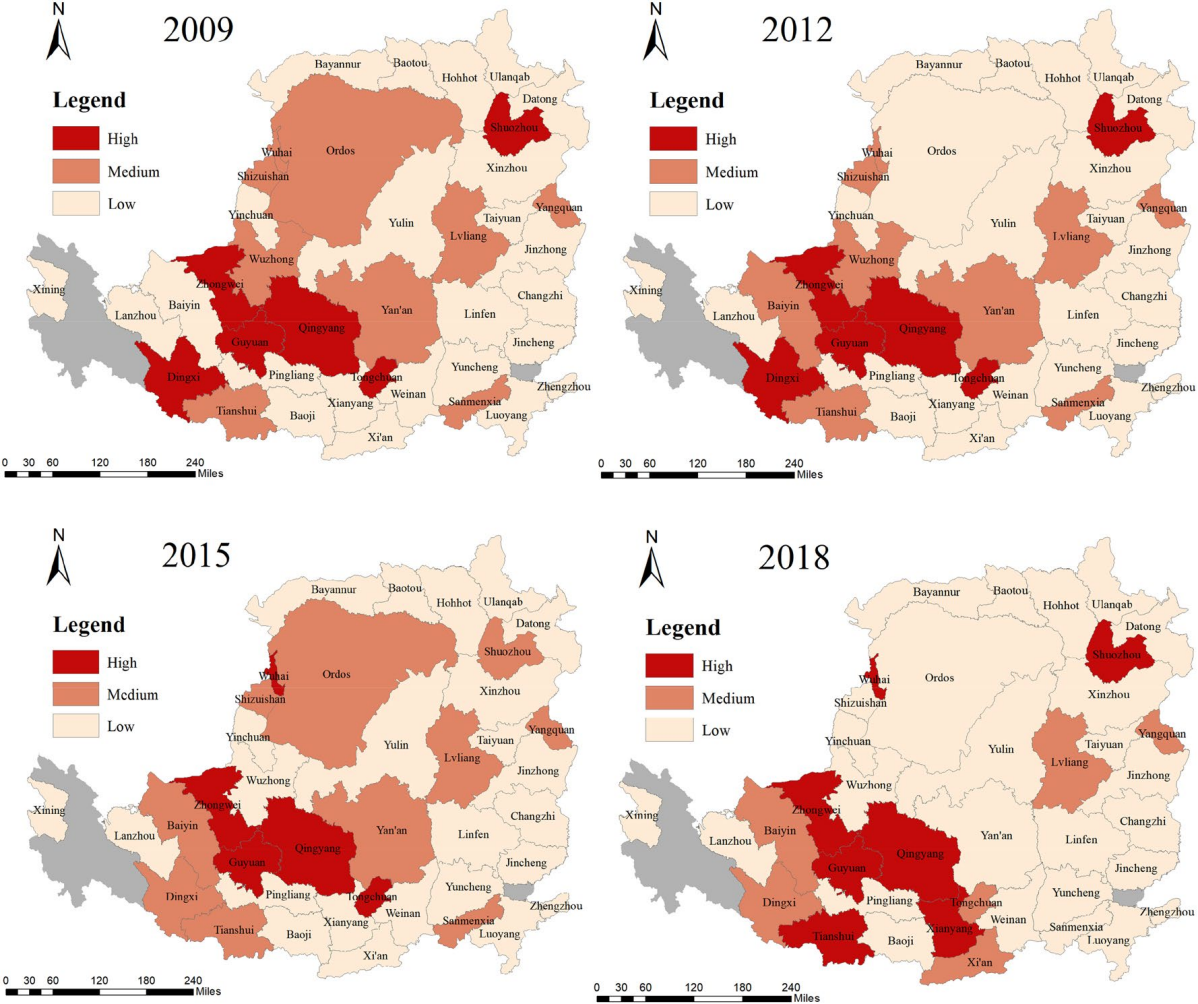
Spatial–temporal evolution of urban LUEE in the study area (Mapping based on the ArcGIS10.8 software can be obtained from the following link, https://desktop.arcgis.com).

**Figure 5:**
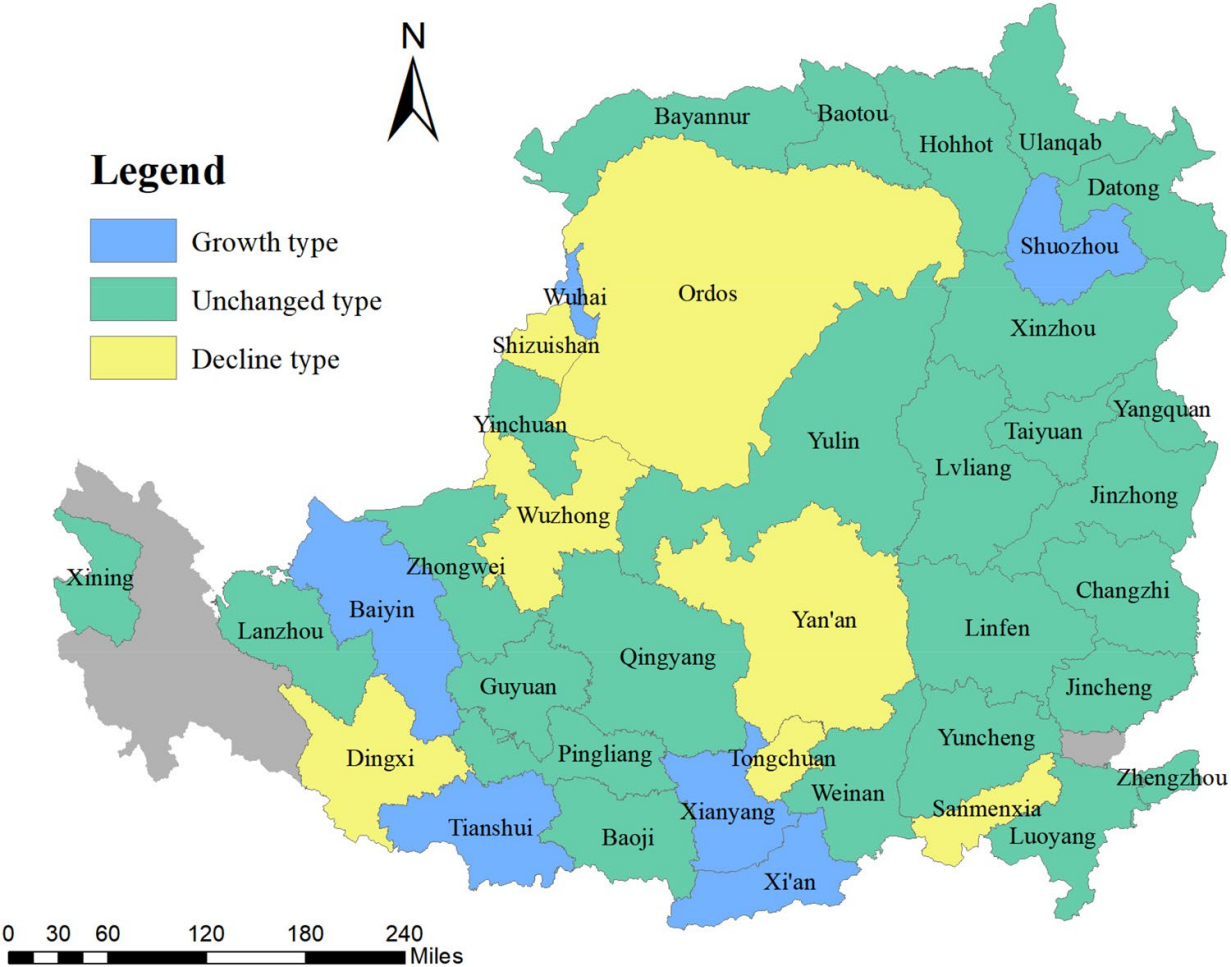
The three types of urban LUEE evolution in the study area (Mapping based on the ArcGIS10.8 software can be obtained from the following link, https://desktop.arcgis.com).

Based on the evaluation results of urban LUEE, the ArcGIS software was used for spatial visualization drawing of urban LUEE in 2009, 2012, 2015, and 2018. It can be found that the cities with high evaluation scores are located in the southwest areas, cities with low assessment level are distributed in the north and east of the study area (Fig. 4). From 2009 to 2018, the LUEE level of most cities in the study area was not ideal, frequent natural disasters and ecological degradation directly restricted the LUEE of the Loess Plateau. How to promote LUEE has become the main content of promoting the efficient development of the research area. It could be found from Fig. 5 that there are three types of LUEE evolution in the Loess Plateau from 2009 to 2018, involving growth type, decline type, and unchanged type. The growth type cities are concentrated in southwest region of the study area, including Wuhai, Shuozhou, Baiyin, Tianshui, Xianyang, Xi’an. The decline type cities are mainly concentrated in the middle, involving Ordos, Shizuishan, Wuzhong, Yan’an, Dingxi, Tongchuan, Sanmenxia. In addition, there are twenty-six cities showed the unchanged trend in urban land use ecological efficiency, accounting for 66.67%. Based on existing studies of the Loess Plateau, it could be found that the ecology of the central region is relatively fragile, the possible reason is that these areas have long faced ecological threats of soil erosion, desertification, and landslides, the comprehensive level of ecological complex system shows a relatively slow growth trend, which makes the urban sustainable construction land resources relatively limited^42,43^.

### Analysis of key factors

From what has been discussed above, the regression results of the urban LUEE in the Loess Plateau can be obtained (Table 4). It could be found that five variables passed the significance test, including PCL, GG, PS, NBP, and REI. Firstly, PCL passed the significant test of 1% with negative coefficient (− 0.1786), indicating the increase of construction land will inhibit the improvement of urban LUEE. Because the sustainable use of urban land resources in the Loess Plateau is limited, the unreasonable planning of urban buildings will occupy a lot of land resources. Secondly, GG passed the significant test of 5% with negative coefficient (− 0.0669), indicating the improvement of urban economic growth rate is not conducive to the optimization of urban LUEE. Excessive pursuit of economic growth will lead to neglect of ecological environment protection. Thirdly, PS passed the significant test of 10% with negative coefficient (− 0.1324), it shows that promoting the development of the secondary industry will hinder the growth of urban LUEE. Industrial activities will pose a threat to air quality, water environmental quality, and soil environmental quality, and the discharge of many industrial pollutants will reduce the comprehensive quality of urban ecological environment. Fourthly, NBP passed the significant test of 1% with positive coefficient (0.1461), indicating the optimization of public transport contributes to the improvement of urban land use ecological efficiency. The development of public transport will reduce urban carbon emissions and improve urban public service capacity. Fifthly, REI passed the significant test of 1% with negative coefficient (− 0.1002), demonstrating the expansion of real estate development scale will inhibit the optimization of land resources. Due to the limitation of landform and the threat of natural disasters, the available land resources of most cities on the study area are insufficient. Real estate development will occupy scarce land resources in most cities of the study area, and how to implement sustainable urban expansion and construction according to local conditions to meet people's basic living conditions is the key content of urban LUEE. Finally, the PRE coefficient is greater than 0, indicating that the impact of technology innovation input on urban land use ecological efficiency is significant. The possible reason is that technological innovation elements can achieve carbon reduction and lower pollutant emissions by improving production efficiency and achieving cleaner production processes^44,45^.

**Table 4:** Regression results based on Tobit model.

Variable	Number	Coefficient	Standard error	T value	P value
Natural population growth rate	*X1*	0.0437	0.0271	1.6100	0.1070
Proportion of urban construction land in urban area	*X2*	− 0.1786	0.0178	− 10.0400	0.0000
Regional GDP growth rate	*X3*	− 0.0669	0.0309	− 2.1600	0.0310
Proportion of secondary industry	*X4*	− 0.1324	0.0341	− 6.2700	0.0780
The number of buses per 10,000 people	*X5*	0.1461	0.0307	4.7600	0.0000
Centralized treatment rate of sewage treatment plant	*X6*	0.0534	0.0898	0.5900	0.5530
Urban real estate investment	*X7*	− 0.1002	0.0166	− 6.0400	0.0000
The proportion of R&D expenditure	*X8*	0.0329	0.0286	1.1500	0.0410
Constant term		1.4092	0.4780	2.9500	0.0030

Additionally, three variables failed the significance test, including PG and CTR. For one thing, it can be found that the coefficient of PG is positive, but it fails the significance test, indicating that urban population growth on the study area has little impact on urban LUEE. The possible reason is that the current population growth in the study area has not brought significant improvement in population quality and increase in labor force, which makes the economic and environmental effects brought by population growth relatively limited^46,47^. For another, the coefficient of CTR is positive, but it fails the test, demonstrating that the effect of urban sewage treatment on urban LUEE is not significant. This is mainly due to the relatively low proportion of urban sewage discharge to urban ecological environment pollutant discharge.

## Discussion

### Urban construction and urban LUEE

**Figure 6:**
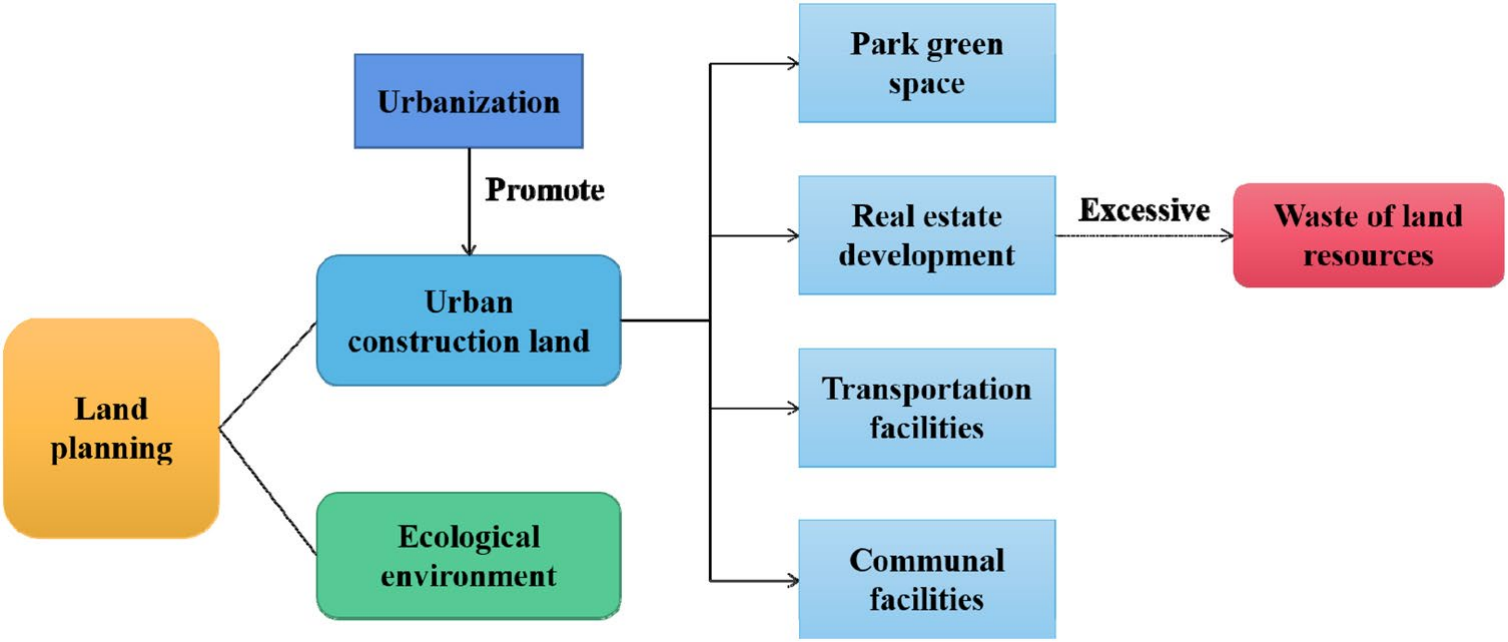
Urban construction and land use/planning.

Through the analysis of the current situation of urban LUEE on the Loess Plateau and the study of key factors, it can be found that there is a complex relationship between urban LUEE and urban construction (Fig. 6). In the study of the relationship between urbanization process and urban construction land, scholars believe that rapid urbanization has changed the original nature of land resources^48,49^. Constrained by natural conditions, the study area is facing the threat of ecological degradation and natural disasters, coordinating the relationship between urban development and urban LUEE is the core element of land planning in these cities^50,51^. Additionally, the real estate development has hindered the optimization of urban LUEE in the study area from 2009 to 2018. Real estate development is the key to urban construction, the environmental practice and green environmental protection measures of real estate enterprises are the key measurements to avoid environmental pollution in the process of engineering construction^52^. The unreasonable expansion of real estate scale will hinder the development of urban LUEE, and the matching of urban real estate construction with residents' needs is one of the directions of building a livable city^53^. Considering the limited resource environmental carrying capacity of some cities in the study area, these cities with scarce land resources are not suitable for large-scale real estate construction and mega-large engineering construction.

### Industrial structure and urban LUEE

**Figure 7:**
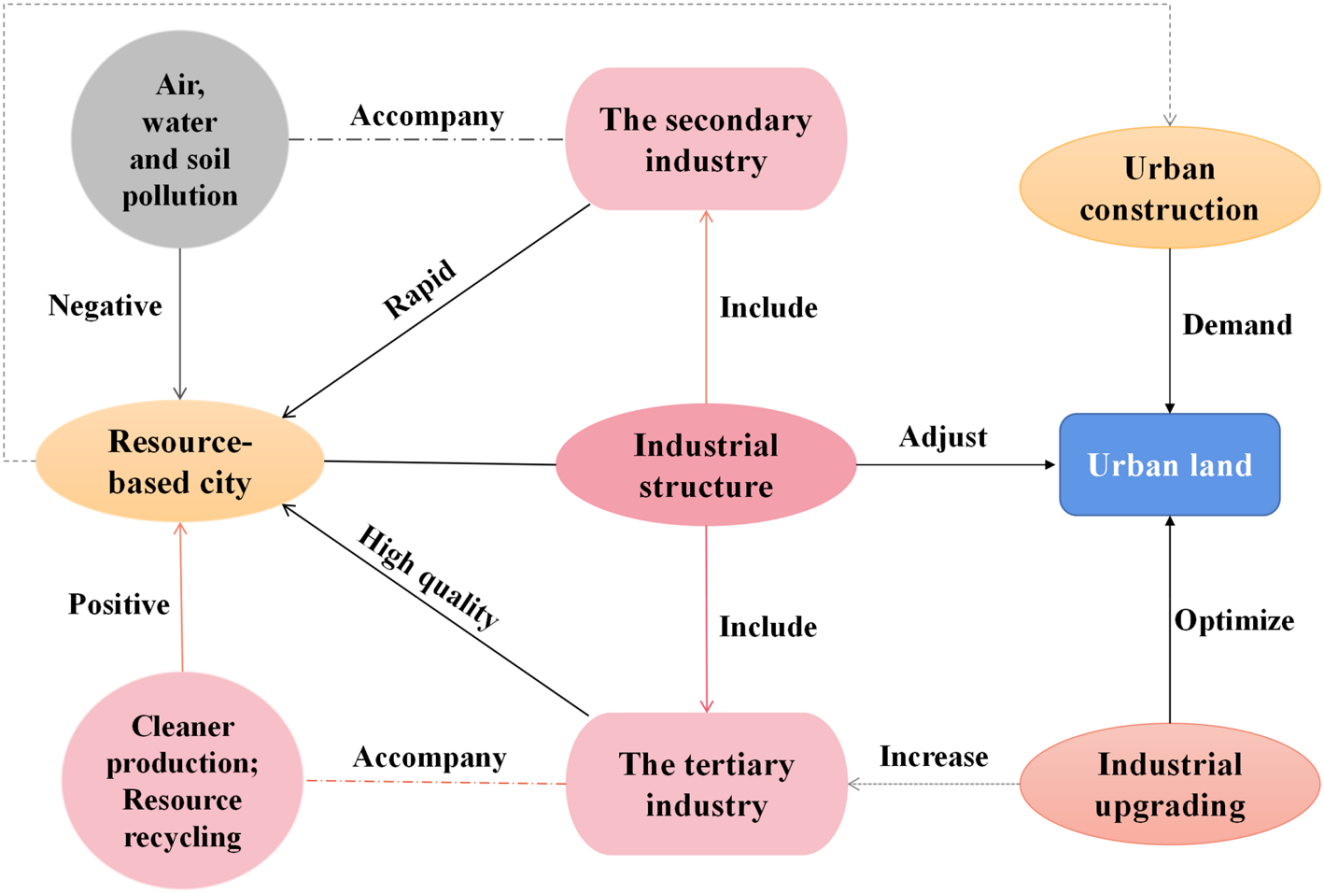
Industrial structure and land use.

The harmonious progress of social development and ecological environment is the focus of existing researches^54,55^. There are many resource-based cities in the study area, whose development and industrial optimization level are relatively backward. These cities may neglect the protection and governance of environment and ecology in the process of pursuing urban economic growth (Fig. 7). However, the development power of resource-based cities comes from relatively single heavy industry, which makes the environmental pollution problems of these cities prominent^56^. The implementation of new urbanization and sustainable development planning makes the adjustment of urban industrial structure the key to the high-quality development of regional cities^57^. The impact of industrial structure, type and land scale on land use is greater than that of policy intervention, and the increase of the proportion of the secondary industry may be unfavorable to urban LUEE^58^. The organic combination of government intervention and urban industrial structure optimization is the key to realize urban LUEE improvement.

### Urban public transportation and urban LUEE

**Figure 8:**
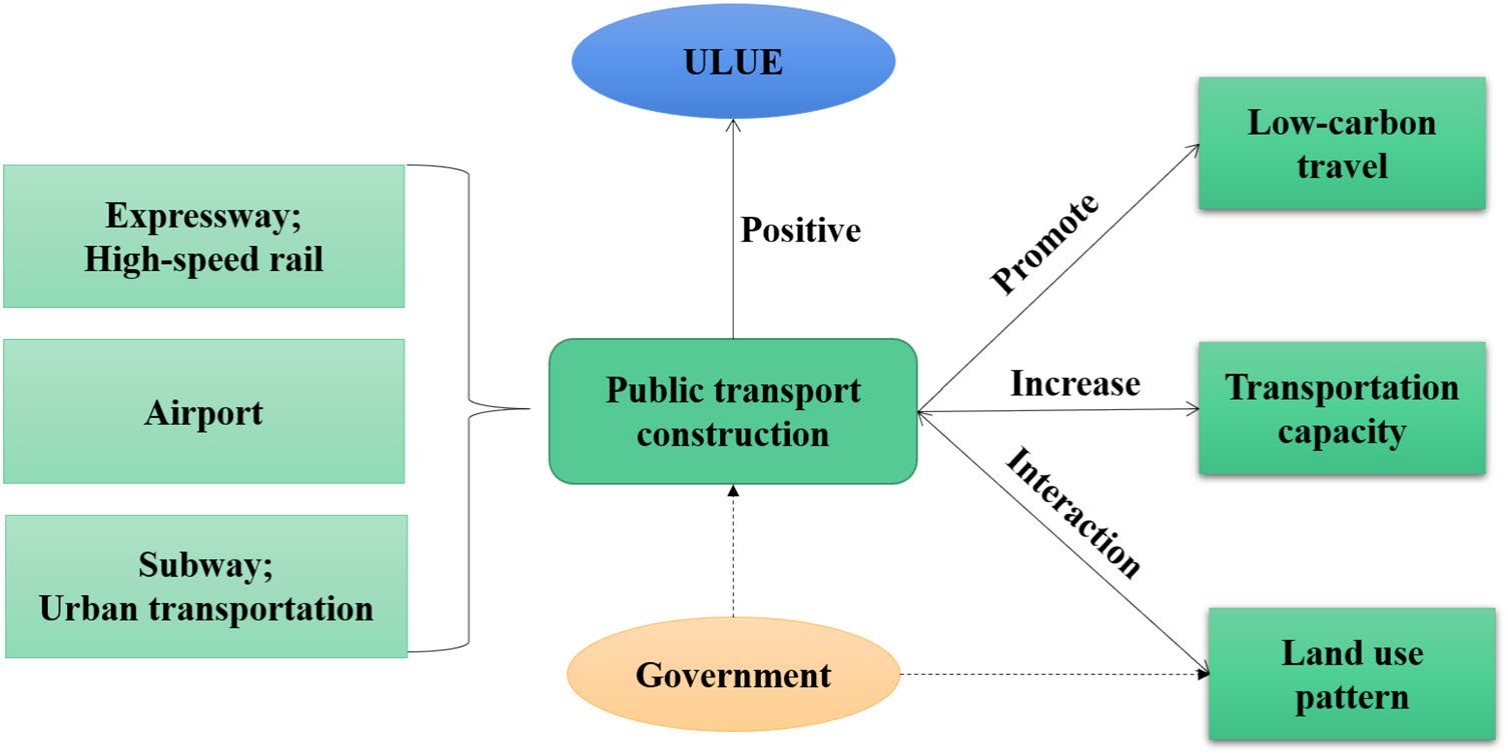
Urban public transportation and land use.

It can be found that the urban public transportation development can significantly promote the urban LUEE (Fig. 8). Relevant researches show that the construction of public transport network is beneficial to urban LUEE. The improvement of land use efficiency can also optimize public transport network and improve the service capacity^59,60^. Due to the improvement of urban public transport network and service functions, low-carbon travel behavior of residents has been significantly affected, which is conducive to reducing the urban air pollution caused by traffic congestion^61^. Additionally, most cities of the study area belong to semi-arid regions, which have faced the problem of insufficient domestic and industrial water resources for a long time^62^. How to recycle river water and rainfall to solve the shortage of urban water resources is of great importance for these cities.

### Policy recommendations

**Figure 9:**
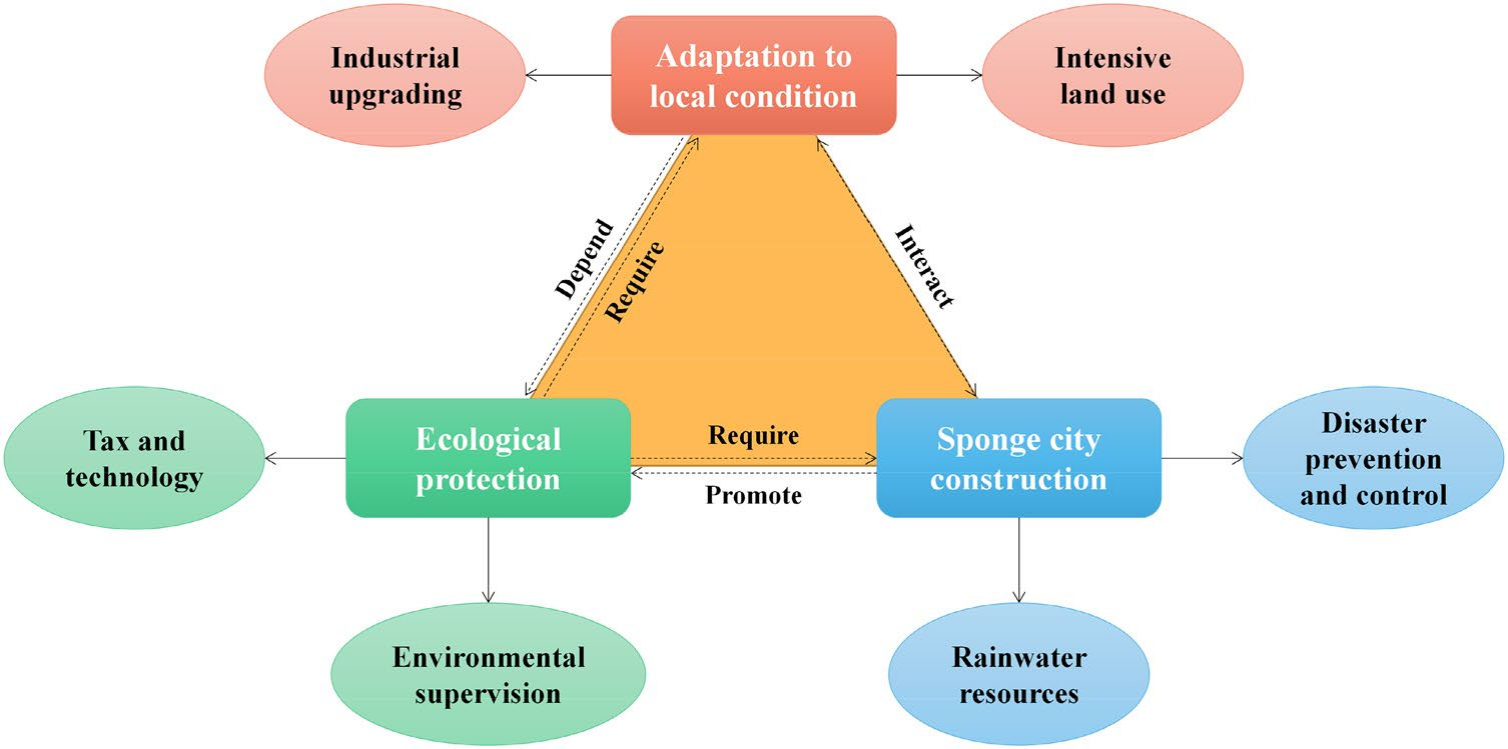
Policy recommendations proposed of this study.

From what has been discussed above, three policy recommendations are proposed for improving the urban land use ecological efficiency and optimizing urban land use management in ecologically sensitive areas, the specific countermeasures are as follows (Fig. 9): (1) Optimizing urban planning. Urban planning in the Loess Plateau is often limited by natural conditions such as terrain and climate, so more attention should be paid to the scientific and sustainable urban planning. Urban functional zoning should be rationally planned to avoid over-development and construction, while strengthening the construction of urban transportation, water conservancy, energy and other infrastructure to improve the carrying capacity and development potential of the city. (2) Strengthening urban land resource management. The management of urban land resources is essential to improve the efficiency of land use. It is necessary to establish and improve the land resource management system, strengthen the monitoring and evaluation of land resources, strictly control the use and development intensity of land resources, and prevent the waste and abuse of land resources. (3) Promoting urban renewal and redevelopment. The old urban areas of the Loess Plateau often have problems such as low land use efficiency and aging infrastructure, and the government should actively promote urban renewal and redevelopment. Through demolition, reconstruction and other ways, the old city can be re-planned and constructed, and modern urban elements and cultural symbols can be introduced to improve the quality and competitiveness of the city.

## Conclusions

This study constructed an index system containing input elements, output elements, and undesired output elements. And it used the super-SBM model to analyze the urban LUEE of 39 cities in the study area from 2009 to 2018. Based on the exploration of temporal-spatial evolution characteristics of urban LUEE and Tobit model, the key factors of urban LUEE were analyzed in ecologically sensitive areas. The study found that in 2009–2018, the time evolution characteristics of urban LUEE presented a "W" upward trend curve, and the spatial characteristics showed an unbalanced development trend with significant spatial differences, the cities with high evaluation scores were located in the western areas and southwestern areas of the study area, the cities with low evaluation scores were located in the north and east areas.

Based on the exploration of urban LUEE key factors in the Loess Plateau from 2009 to 2018, the key factors were extracted by Tobit model and empirical analysis. There were five variables that have a significant effect on urban LUEE. This study provides an evaluation index system of urban LUEE, which can be used for land research in ecologically sensitive areas or other similar areas. Based on the discussion of key factors, the driving factors of urban ecological environment protection and urban social and economic sustainable development can be found. This research is an indicator system constructed through the analysis and summary of previous surveys, and there is still room for further improvement. How to ensure the scientific nature of the indicator system is the direction of future research.

## Data Availability

The datasets used and analyzed during the current study available from the corresponding author on reasonable request.
